# Prosthetic Aortic Valve Endocarditis Caused by Burkholderia cepacia complex: A Case Report

**DOI:** 10.7759/cureus.89793

**Published:** 2025-08-11

**Authors:** Khawar Tariq Mehmood, Amina Shahid

**Affiliations:** 1 Internal Medicine, Aster Hospital, Branch of Aster DM Healthcare-A Free Zone Company (FZC), Dubai, ARE; 2 Internal Medicine, New Medical Centre (NMC) Speciality Hospital Al Nahda, Dubai, ARE

**Keywords:** burkholderia cepacia, endocarditis, gram-negative endocarditis, multi-drug resistance, prosthetic aortic valve

## Abstract

Prosthetic valve endocarditis (PVE) is a rare condition caused by infection of prosthetic heart valves (PHV) or repaired native valves. Onset from the time of implantation can be used to classify it into early and later categories. Gram-positive cocci are the predominant cause of early PVE (less than one year after implantation), though other less common organisms can also cause PVE. We report a case of early prosthetic valve endocarditis caused by gram-negative *Burkholderia cepacia* complex (BCC). This multidrug-resistant organism is an exceedingly rare cause of PVE. Only a handful of cases have been reported in medical literature. Its inherent resistance to the commonly used treatment modalities presents a unique challenge. Often, surgical intervention and removal of the prosthesis are required to ensure resolution. We present a case of prosthetic aortic valve endocarditis caused by *Burkholderia cepacia* (*B. cepacia*) that showed an excellent response to combination antibiotics.

## Introduction

Infective endocarditis (IE) is an infection of the native heart valves, prosthetic heart valves (PHV), or endocardial surface and is associated with significant morbidity and mortality [1]. Prosthetic valve endocarditis (PVE) accounts for 1% to 5% of all cases of IE and carries mortality rates as high as 23% [2]. The gram-positive coccus *Staphylococcus aureus* is the most frequently isolated organism, but other organisms, such as coagulase-negative *Staphylococcus*, *Streptococcus*, and *Enterococcus*, are also common causes [2].

*Burkholderia cepacia* (*B. cepacia*) is an exceedingly rare cause of PVE, with only a handful of cases reported in the medical literature [3]. *B. cepacia* is a gram-negative bacillus that is resistant to multiple classes of antibiotics, including beta-lactams, fluoroquinolones, and trimethoprim, as well as some disinfectants [4,5]. This bacterium is typically seen in immunocompromised patients, intravenous drug users (IVDUs), and individuals with prosthetic valves [6]. The rarity of this etiological agent, combined with its antibiotic resistance, poses a significant challenge to effective treatment.

We present a case of an immunocompetent individual who developed prosthetic aortic valve endocarditis caused by a *Burkholderia *species, successfully managed with combination antibiotic therapy, resulting in an excellent outcome. We also review the literature on this uncommon infection.

## Case presentation

A 55-year-old male patient was admitted to our facility with a two-week history of fever, fatigue, and shortness of breath. His symptoms had developed gradually and progressively worsened over the past two weeks. The fever was high-grade (maximum recorded temperature: 103.2°F), intermittent, associated with chills, and temporarily relieved with antipyretics. He also reported increasing lethargy and exertional dyspnea. The shortness of breath had an insidious onset, was progressively worsening, and was now occurring during routine daily activities. There was no history of cough, wheezing, chest pain, orthopnea, or paroxysmal nocturnal dyspnea. He denied any weight loss, change in appetite, lymphadenopathy, skin rash, headache, sore throat, nausea, vomiting, altered bowel habits, joint pain, dysuria, or hematuria. He reported no history of smoking, alcohol consumption, or IVDU. 

Six months prior, he had undergone aortic valve replacement with a St. Jude Medical HP™ 21 mm mechanical prosthetic valve (Abbott Laboratories, Abbott Park, IL) for symptomatic aortic stenosis. He was on oral anticoagulation (warfarin) and a beta-blocker (nebivolol). However, he had self-discontinued warfarin following the onset of his symptoms (last international normalized ratio (INR) prior to presentation: 2.4). He had also completed a five-day outpatient course of amoxicillin-clavulanic acid and ciprofloxacin without symptomatic improvement, prompting his hospital presentation.

On examination, he was hemodynamically stable. His vital signs were as follows: blood pressure of 113/67 mmHg, pulse rate of 90 beats per minute, respiratory rate of 20 breaths per minute, temperature of 100.2°F, and oxygen saturation of 99% on room air. Cardiac examination revealed an ejection systolic murmur (Grade 2/6) at the right second intercostal space. Systemic examination was otherwise unremarkable, and there were no peripheral stigmata of IE.

The patient's initial laboratory investigations are summarized in Table 1.

**Table 1:** A Summary of Important Investigations of the Patient

Parameter	Value	Reference Range
Total Leucocyte Count	8.8 ×10^3 ^/µL	4-11 × 10^3 ^/µL
Hemoglobin	10.8 g/dl	13-18 g/dl
Platelet count	218 ×10^3 ^/µL	150-450 ×10^3 ^/µL
Procalcitonin	0.672 ng/ml	<0.1 ng/ml
Erythrocyte Sedimentation rate	28 mm/hour	0-15 mm/hr
C-reactive Protein	110.73 mg/dl	<1 mg/dl
Troponin	115.9 pg/ml	<14 pg/ml
International Normalized Ratio	1.676	0.8-1.2
Alanine Transaminase	46 U/L	7-56 U/L
Aspartate Aminotransferase	39 U/L	8-33 U/L
Total Bilirubin	1.56 mg/dl	0.1-1.2 mg/dl
Albumin	3.64 gm/dl	3.5-5.5 gm/dl
Protein	6.7 gm/dl	6-8.3 gm/dl
Alkaline Phosphatase	71 U/L	30-130 U/L
Creatinine	1.03 mg/dl	0.7-1.3 mg/dl
Hemoglobin A1c	5.40%	<5.7%

Transthoracic echocardiography (TTE) revealed a prosthetic metallic aortic valve in situ with an echogenic structure on the ventricular aspect of the valve, suggestive of vegetation. There was a significant transvalvular pressure gradient (peak: 75 mmHg; mean: 41 mmHg).

Therapeutic anticoagulation with enoxaparin (1 mg/kg every 12 hours) and beta-blockers was initiated. Four sets of blood cultures (collected eight hours apart from four different sites) were obtained. Transesophageal echocardiography (TEE) was advised for better characterization of the vegetation, but was declined by the patient due to its invasive nature and personal preference.

All four blood culture sets were positive for gram-negative bacilli, and the patient was started empirically on ceftriaxone and gentamicin. Subsequent culture reports confirmed *B. cepacia* in all four samples, with sensitivities as shown in Table 2.

**Table 2:** Antimicrobial Susceptibility of Isolated Burkholderia cepacia complex R: Resistant; I: Intermediate; S: Sensitive; MIC: Minimum Inhibitory Concentration (µg/ml)

Antibiotic Name	Interpretation	MIC (µg/ml)
Ceftazıdıme	I (Intermediate)	16
Co-trimoxazole	S (Sensitive)	≤1/19
Levofloxacın	S (Sensitive)	≤1
Meropenem	S (Sensitive)	4

The patient satisfied both of the modified Duke's major criteria and was diagnosed as a case of PVE due to *B. cepacia*. Based on sensitivity testing, the antibiotic regimen was adjusted to meropenem and levofloxacin. Trimethoprim-sulfamethoxazole was later added following consultation with an infectious disease specialist.

The patient demonstrated clinical improvement with treatment. Fatigue and dyspnea lessened, fever spikes resolved, and serial C-reactive protein (CRP) levels showed a declining trend. Follow-up blood cultures, obtained one week after initiating antibiotics, were negative. Warfarin was restarted for anticoagulation, and the INR was optimized. The patient was discharged following resolution of fever and a negative blood culture, with instructions to continue an outpatient antibiotic regimen consisting of meropenem 2 g IV every eight hours for six weeks, levofloxacin 500 mg orally twice daily for 12 weeks, and trimethoprim-sulfamethoxazole 800/160 mg (two tablets) orally twice daily for four weeks.

The duration of treatment was calculated from the date of the first negative blood culture. He remained asymptomatic during outpatient follow-up. Serial echocardiograms showed improvement in the transvalvular gradient and reduction in vegetation size. He remained well at his six-month follow-up with his cardiologist, and repeat echocardiography confirmed complete resolution of vegetation and further improvement in the transaortic pressure gradient (peak gradient of 50 mmHg and a mean gradient of 27 mmHg).

Our case highlights a rare cause of PVE that responded well to medical management, avoiding the need for surgical intervention.

## Discussion

PVE is an uncommon but potentially serious cause of IE, with mortality rates approaching 20% to 30% [2]. PVE is defined as an endovascular, microbiological infection that occurs on the surface of a PHV or reconstructed native heart valve [7]. The reported frequency of occurrence ranges from 0.1% to 2.3% per patient year [8-10]. The presence of foreign material predisposes to infection with coagulase-negative, novobiocin-susceptible *Staphylococci *due to their ability to adhere to a variety of materials [11,12]. Time of onset following surgery is used to classify PVE into early (within one year of surgery) and late (after one year of surgery) categories with important differences in mode of transmission and etiological agents [9,11,13]. Mechanical valves are more susceptible to infection compared to bioprostheses during the early post-implantation period [12,14]. Conversely, late PVE seems to be more common in bioprosthetic valves [7]. The etiological microbes also differ between early- and late-onset PVE. Gram-positive organisms such as *Staphylococcus aureus*, *Staphylococcus epidermidis*, and fungi are the predominant causes of early PVE [15]. At the same time, the incidence of enterococci and *Streptococcus viridans* infection increases in late-onset PVE [15]. Numerous preventive strategies are available to reduce the risk of PVE. Administration of surgical antimicrobial prophylaxis (SAP) closer to the time of surgical incision is associated with lower rates of postoperative infections [16-18]. Some studies show promise of using antimicrobial-impregnated prostheses to reduce the risk of bacterial adherence and consequent infection [19,20]. Stringent asepsis and good surgical technique are essential in preventing early PVE [21]. Late PVE can be prevented with regular mouth care and appropriate preprocedural prophylactic antibiotics [21].

Diagnosis of PVE may be challenging due to atypical presentation, the presence of extracardiac features, and complications [7]. The validated Duke’s criteria, which are used for the diagnosis of native valve endocarditis (NVE), may have less sensitivity for the diagnosis of PVE [7,22]. Nevertheless, echocardiographic features and microbiological isolation form an important basis for diagnosis. Three echocardiographic features are particularly sensitive for PVE: vegetation attached to PHV, evidence of abscess or fistula, and new dehiscence of a PHV [22]. TTE should be performed if the transthoracic echo is negative. Guidelines for microbiological isolation recommend withdrawal of at least three sets of blood cultures within the first 24 hours of admission, spaced at least one hour apart [22,23].

*B. cepacia *and PVE

*B. cepacia complex* (BCC) is a group of several genetically different but phenotypically similar bacteria [24]. This organism is classically associated with pulmonary disease, especially in patients with cystic fibrosis, but it has also been known to cause granulomatous infections and affect immunocompromised individuals [25]. NVE caused by *Burkholderia *spp. has been reported, with IVDU being an important risk factor [26-28]. PVE by this organism is exceedingly rare, with only a handful (around 19) of case reports published in English literature. 

Table 3 below summarizes previously documented cases of PVE caused by BCC.

**Table 3:** Summary of Documented Cases of Burkholderia cepacia-Associated Prosthetic Valve Endocarditis

S. no.	Author	Patient Age (years)	Gender	Valve Involved	Antibiotic Regimen	Surgical Intervention	Survival
1.	Gonzalez et al. [3]	37	Male	Mitral and Nativetricuspid Valves	Ceftazidime, Meropenem, Trimethoprim-Sulfamethoxazole	Yes	Yes
2.	Noreiga et al. [26]	42	Male	Aortic Valve	Sulfamethoxazole-Trimethoprim-Polymyxin	Yes	No
3.	Noreiga et al. [26]	25	Male	Mitral Valve	Sulfamethoxazole-Trimethoprim-Polymyxin	Yes	Yes
4.	Dellana et al. [29]	38	Male	Mitral Valve	Ceftazidime, Vancomycin, Levofloxacin	Yes	Yes
5.	Speller [30]	55	Male	Mitral Valve	Trimethoprim-sulfamethoxazole, Kanamycin	No	No (From an Unrelated Infection)
6.	Speller [30]	48	Female	Mitral Valve	Trimethoprim-Sulfamethoxazole, Kanamycin	No	Yes
7.	Rahal et al. [31]	-	-	Tricuspid Valve	Trimethoprim-Sulfonamide, Polymyxin	Yes	No
8.	Bhojraj et al. [32]	54	Female	Mitral Valve	Piperacillin and Tazobactam, Then 30 Days Later, Imipenem and Cilastin	No	No
9.	Bhojraj et al. [32]	71	Male	Aortic Valve	Meropenem	No	No
10.	Russo et al. [33]	75	Female	Mitral Valve	Cefepime, Trimethoprim-Sulfamethoxazole	Yes	Yes
11.	Aggarwal et al. [34]	58	Female	Mitral Valve	Trimethoprim-Sulfamethoxazole	-	-
12.	Balaji et al. [35]	28	Female	Mitral Valve	Trimethoprim-Sulfamethoxazole, Levofloxacin, Ceftazidime	Yes	Yes
13.	Durate-Mangoni et al. [36]	56	Male	Mitral Valve	Imipenem, Vancomycin	Yes	Yes
14.	Saraswaat et al. [37]	47	Male	Aortic Valve	Caspofungin, Cefepime	No	Yes
15.	Nnaoma et al. [38]	32	Male	Mitral Valve	Vancomycin, Cefepime, and Later Levofloxacin	Yes	Yes
16.	Chipigina et al. [39]	34	Female	Mitral Valve	Trimethoprim-Sulfamethoxazole	No	No
17.	Moy et al. [40]	56	Male	Mitral Valve	Meropenem, Levofloxacin, Trimethoprim-Sulfamethoxazole, Later Meropenem Switched to Ceftazidime-Avibactam	No	No
18.	Singh et al. [41]	69	Female	Aortic Valve	Vancomycin, Gentamicin, and Rifampicin	Yes	No
19.	Present Case	55	Male	Aortic Valve	Trimethoprim-Sulfamethoxazole, Meropenem, Levofloxacin	No	Yes

**Figure 1:**
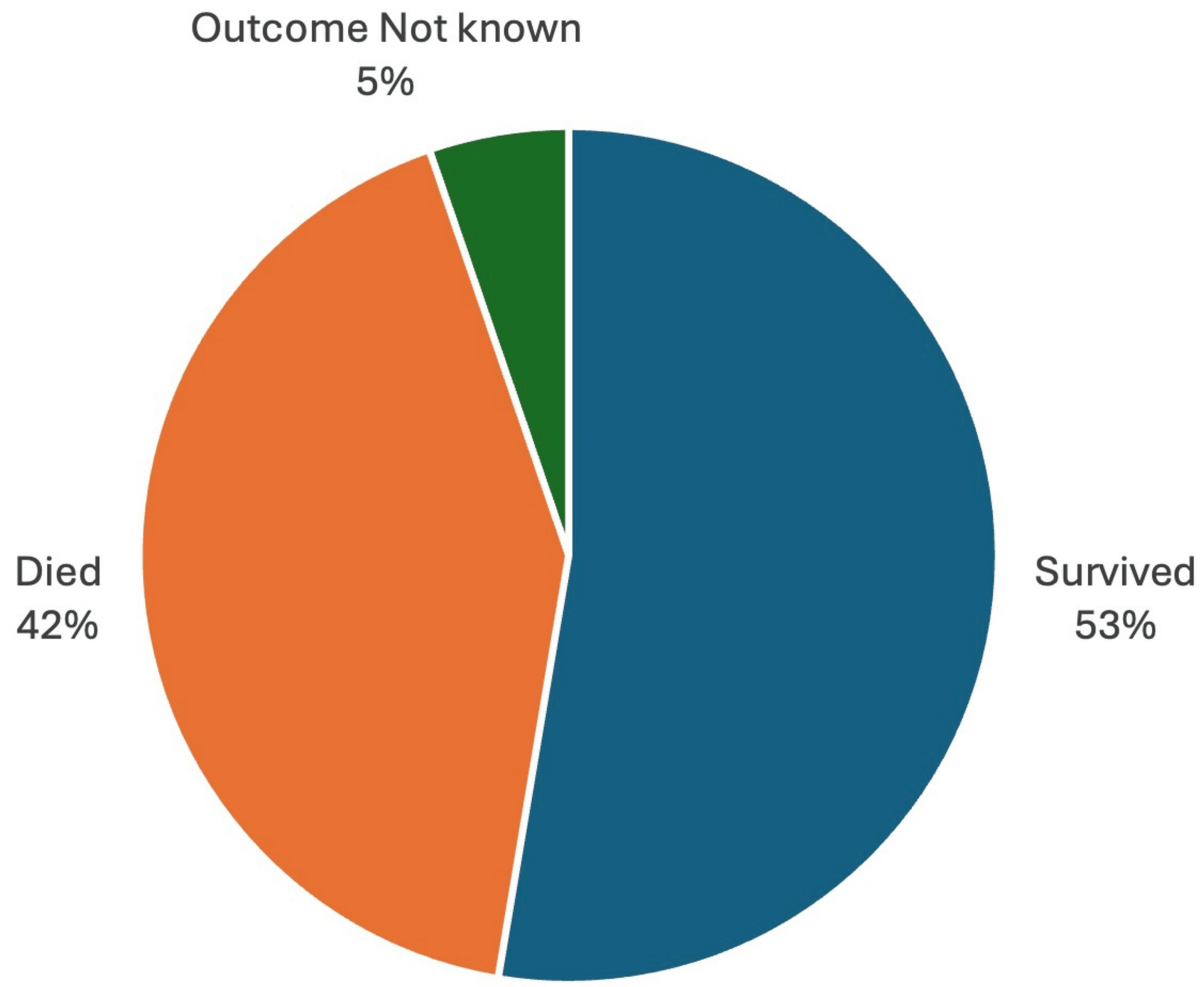
Mortality in Prosthetic Valve Endocarditis Caused by Burkholderia cepacia complex Note: This Image Has Been Created by the Authors.

**Figure 2:**
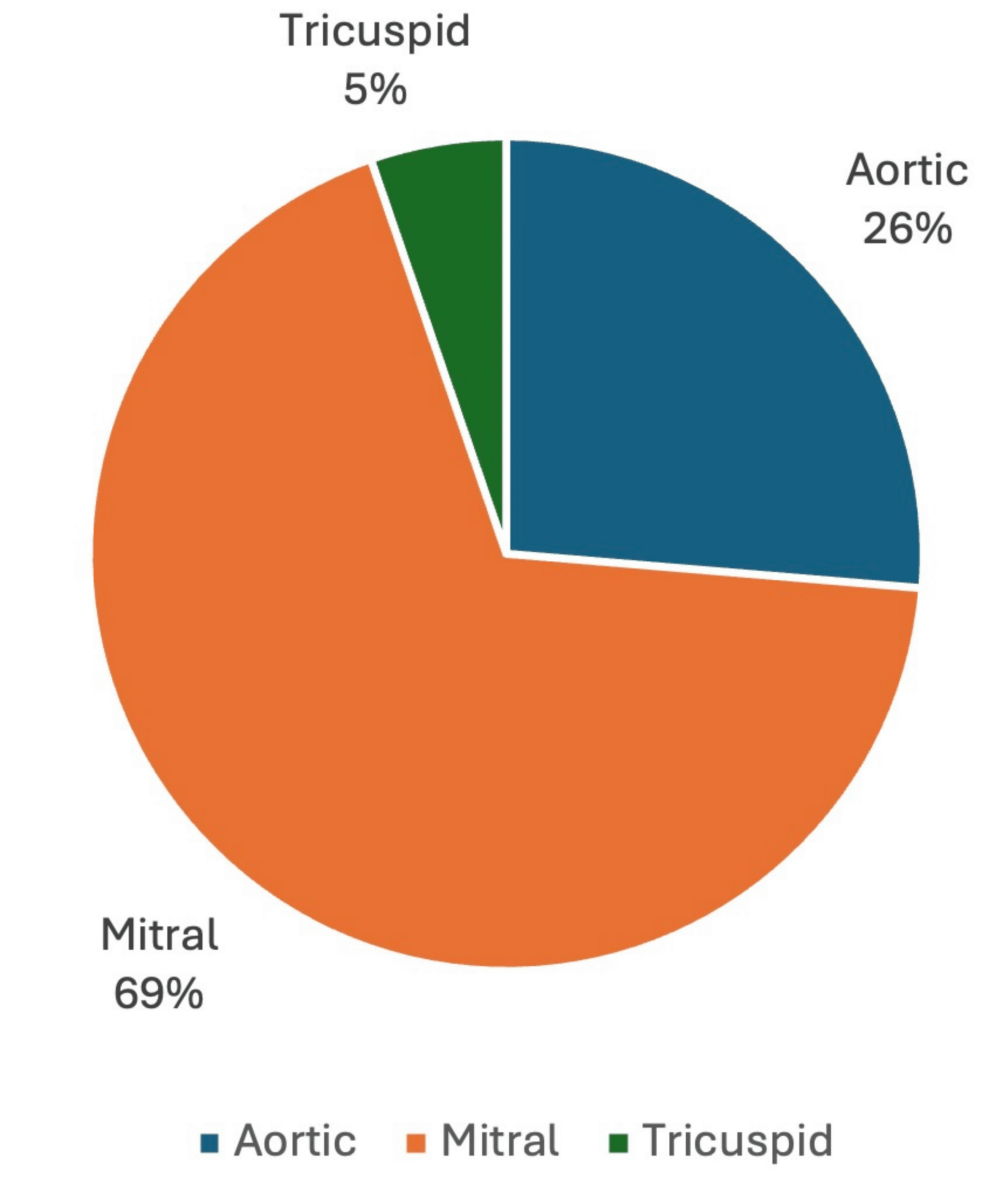
Frequency of Affected Valvular Prosthesis by Burkholderia cepacia complex Note: This Image Has Been Created by the Authors.

**Figure 3:**
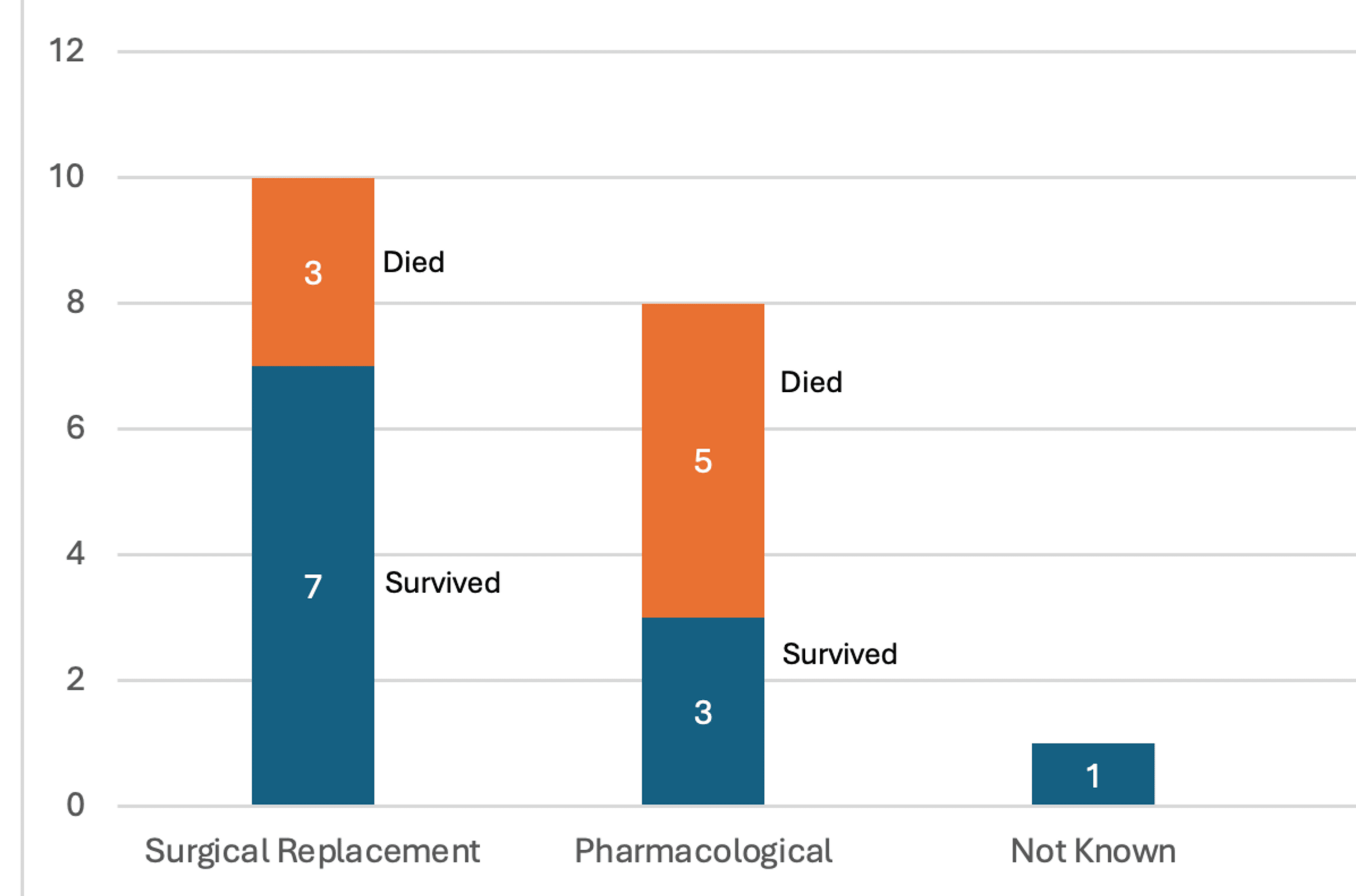
Treatment Modalities Used For Cases of Burkholderia cepacia complex Prosthetic Valve Endocarditis Note: This Image Has Been Created by the Authors.

A review of the literature shows that PVE caused by *Burkholderia *has a high mortality (approaching 42%) (Figure 1). Difficulty isolating the organisms and inherent resistance to first-line treatment for endocarditis may play a role in high mortality. The organisms’ propensity to affect prosthetic mitral valves (69%), followed by aortic prosthetic involvement (26%), may be due to the higher number of mitral prostheses compared to other valves (Figure 2). More than half of the reported cases required surgical correction, and the remainder were managed with combination pharmacotherapy (Figure 3) [1-42].

BCC is inherently resistant to multiple antimicrobial agents [5]. A study on antimicrobial sensitivity conducted by Zhou et al. showed almost 50% of the isolates were resistant to commonly used antibiotics such as chloramphenicol, trimethoprim-sulfamethoxazole, ciprofloxacin, tetracycline, rifampin, avibactam, and amoxicillin-clavulanate [42]. However, this study may have overestimated the resistance in *Burkholderia *spp., as it was conducted on isolates known to be multidrug-resistant. A case report and literature review conducted by Dellalana et al. showed that trimethoprim-sulfamethoxazole was the mainstay of therapy along with additional agents such as carbapenems, penicillin, quinolones, kanamycin, and polymyxin [29]. A review of published cases also showed trimethoprim-sulfamethoxazole was the most used antibiotic agent, often combined with meropenem or levofloxacin (Table 2).

We used a combination of trimethoprim-sulfamethoxazole, meropenem, and levofloxacin as our treatment regimen, with trimethoprim-sulfamethoxazole forming a key component, as seen in the majority of published cases. The duration of treatment was approximately four to 12 weeks following the first negative blood culture. Due to the unavailability of an intravenous formulation at our facility, we administered trimethoprim-sulfamethoxazole orally.

The patient demonstrated an excellent clinical response to the regimen, with serial examinations showing complete resolution of the echocardiographic valvular lesion. Surgical intervention was not required, owing to the prompt clinical response and absence of complications. It is likely that the patient’s younger age and lack of comorbidities contributed to the favorable outcome in this case. Our case highlights the need for early institution of combination antibiotics that are known to be effective against *B. cepacia*. The combination of trimethoprim-sulfamethoxazole, quinolones, and carbapenem seems to be highly effective against this multi-resistant organism.

Further study is needed to recommend a standardized treatment regimen and duration of therapy. Early institution of effective antibiotics, together with prompt surgical intervention in the presence of complications, is essential to reducing morbidity and mortality.

## Conclusions

This case underscores the clinical significance of BCC as a rare yet formidable cause of PVE. Our patient, an immunocompetent individual with early-onset PVE, demonstrated complete clinical and echocardiographic resolution with a tailored combination of trimethoprim-sulfamethoxazole, meropenem, and levofloxacin without requiring surgical intervention. This successful outcome highlights the potential efficacy of appropriately selected antimicrobial therapy even in the context of multidrug resistance.

Notably, our case adds to the limited literature on *B. cepacia*-associated PVE and offers insights into management strategies that may be considered in similar clinical scenarios. The decision to exclude surgical management, supported by early diagnosis and close clinical monitoring, may have been aided by the patient’s younger age and absence of comorbidities.

Given the paucity of data and the organism’s inherent antimicrobial resistance, future studies should focus on establishing standardized treatment protocols, including the most effective antimicrobial combinations and optimal duration of therapy. There is also a need to evaluate the potential role of novel therapeutic agents and innovative drug delivery systems, such as antibiotic-impregnated prosthetic valves, in managing such infections. Additionally, identifying host-related factors associated with favorable outcomes could help guide more individualized treatment strategies. Finally, the utility of advanced molecular diagnostic techniques should be explored, particularly in cases where traditional cultures fail to identify the pathogen.

As the incidence of prosthetic valve implantation continues to rise, awareness of uncommon pathogens like *B. cepacia* is essential. Enhanced surveillance, timely microbiological diagnosis, and personalized therapy remain central to improving outcomes in this challenging subset of IE.
